# Neighborhood Police Encounters, Health, And Violence In A Southern City

**DOI:** 10.1377/hlthaff.2021.01428

**Published:** 2022-02

**Authors:** Katherine P. Theall, Samantha Francois, Caryn N. Bell, Andrew Anderson, David Chae, Thomas A. LaVeist

**Affiliations:** Tulane University, New Orleans, Louisiana.; Tulane University.; Tulane University.; Tulane University.; Tulane University.; Tulane University.

## Abstract

The disproportionate rates of police surveillance and encounters in many communities in the US may be contributing to inequities in health and violence. Frequent policing in communities, which may often also be aggressive policing, has been associated with diminished health and well-being. This study adds to the growing body of research on this issue by examining the relationships between neighborhood police stop-and-frisk encounters and both health outcomes and violence rates in New Orleans, Louisiana, in an ecological, cross-sectional study using local police report, Centers for Disease Control and Prevention, and census data. The average rate of police stop-and-frisk encounters was more than three times higher for Black adults compared with their White counterparts. Even after we accounted for concentrated disadvantage (a high percentage of residents of lower socioeconomic status) and residential racial and income segregation, neighborhoods with higher rates of encounters had significantly higher prevalence rates of smoking, physical inactivity, and poor physical health, and they experienced significantly more violent crime (18.35 more per 1,000) and domestic violence (49.91 more per 1,000) events than neighborhoods with lower levels of police encounters. There is a need for strengthened policy focused on the relationship between frequent policing and health and violence outcomes.

During the past decade the US has seen an increase in the monitoring, tracking, and reporting of state-sanctioned violence (that is, discriminatory policing) against racially marginalized communities. The previous and most recent high-profile police killings of Black people and the disproportionate rates at which Hispanic, Latino, or Latinx immigrants are detained, arrested, and incarcerated have captured global attention and prompted a movement for change within academic medical and public health research communities.^1^ Disproportionate experiences of police violence and mass incarceration among African Americans or Black Americans are not new problems.^2,3^ However, from the killings of Eric Garner and Michael Brown in 2014 to the killings of Breonna Taylor and George Floyd in 2020, there has been a resurgence of attention on connecting racial disparities in state-sanctioned violence to racist and oppressive systems, structures, and institutions.

In the most recent Police-Public Contact Survey (2018), a nationally representative sample of households and residents ages sixteen and older, 24 percent of respondents indicated experiencing contact with police,^4^ up from 21 percent in the 2015 survey.^5^ Traffic stops were the most-reported encounter, with Black Americans more likely to be the driver in such stops compared with non-Hispanic White and Hispanic or Latinx populations. Black Americans also were more likely to encounter street stops and to report the use of force or being threatened by police. These experiences of increased police contact also start at an early age. For example, nearly 20 percent of youth ages 9–15 in the Fragile Families and Child Wellbeing Study (2014–17) indicated that they had been stopped by the police.^6^ Also, nearly 10 percent of adolescents ages 12–18 in the Panel Study of Income Dynamics Child Development Supplement (2002 and 2007) indicated that they had been stopped two or more times in the past six months.^7^ In a smaller survey of predominantly Black and Latinx boys in the ninth and tenth grades (2014 and 2015), 40 percent of the survey sample experienced at least one police stop in the past six months.^8^

In addition, studies show that living in neighborhoods with more frequent policing (or what is sometimes called “hyperpolicing” or “proactive policing”)—which involves greater stops, searches, and surveillance by police in some communities to detect criminal activity or disrupt circumstances that may lead to criminal activity, and often includes techniques such as “stop-and-frisk”^9-12^—may have negative effects on health outcomes such as self-rated health, depression, anxiety, posttraumatic stress disorder, suicidal ideation, high blood pressure or hypertension, asthma, and obesity.^7,13-19^ Among youth, specifically, these police encounters have been linked to experiences of stress, trauma, and anxiety,^14,20^ including posttraumatic stress symptomatology.^21^

These health outcomes, which do not rely on personal encounters, suggest that people living in frequently policed neighborhoods need not experience contact with the police to be affected by the stress of the events. Even the indirect experiences of violence in one’s community can effect physiologic responses in ways that may increase the risk for cardiovascular conditions and other health outcomes,^22,23^ and for youth, witnessing police stops may be a significant source of emotional distress.^20^

Despite mounting evidence demonstrating the negative effects of frequent policing,^2,24^ there remains a need to quantify associations between police encounters and health and well-being, including community violence.We acknowledge, however, that the link between frequent policing and violence is cyclical and compounding, affecting not only additional potential acts of interpersonal or community violence^8,25,26^ but also physiologic responses that may affect health.^27^ Adopting the conceptual model of Maayan Simckes and colleagues,^27^ we examined the association between frequent policing and both individual (for example, health and behavior) and community-level outcomes, examined at the community level.

Our study examined the relationships between neighborhood police encounters, focusing on stop-and-frisk encounters, for both adult health outcomes (such as mental health and obesity) and violence-related outcomes (including domestic events and the rate of violent crime) in New Orleans, Louisiana. New Orleans had the fourth-highest murder rate in the US in 2019^28^— a rate five times the national average (30.7 per 100,00 versus 5 per 100,000).^29^ Our hypothesis was that frequent policing in Black communities may be associated with the racial health disparities seen across so many communities. Our chief outcome of interest was the rate of police stop-and-frisk encounters or “pat-downs” within a census tract per population in 2018, examined in total as well as separately both for encounters among Black residents and White residents and specifically for youth encounters (per youth population). Our research design allowed us to show the associations between stop-and-frisk encounters and a variety of health-related and violence outcomes by neighborhoods in New Orleans.

## Study Data And Methods

### Data Sources

This secondary, ecologic cross-sectional study was conducted with publicly available data from the City of New Orleans (2018),^30^ the 500 Cities Project (2016–19) of the Centers for Disease Control and Prevention (CDC),^31^ and the American Community Survey (ACS). *Neighborhood* was defined as a census tract,^32^ with the number of tracts included in the study varying based on outcome, including 174 census tracts with available data for violence-related outcomes and 48 tracts with available CDC Behavioral Risk Factor Surveillance System (BRFSS) data.

### Measures

Outcome variables from the CDC’s 500 Cities Project data, based on state BRFSS data,^31^ included (among respondents ages eighteen and older) the prevalence of cigarette smoking (respondents who report having smoked at least 100 cigarettes in their lifetime and who currently smoke every day or some days), coronary heart disease (respondents who report ever having been told by a doctor, nurse, or other health professional that they had angina or coronary heart disease), diabetes (having been told by a doctor, nurse, or other health professional that they had diabetes), obesity (respondents who have a body mass index of at least 30.0 kg/m^2^, calculated from self-reported weight and height), poor mental health (respondents who report fourteen or more days during the past thirty days during which their mental health was not good), poor physical health (respondents who report fourteen or more days during the past thirty days during which their physical health was not good), poor sleep (respondents who report usually getting insufficient sleep—or less than seven hours of sleep, on average, during a twenty-four-hour period), and a lack of leisure time physical activity (respondents who answered “no” to the following question: “During the past month, other than your regular job, did you participate in any physical activities or exercises such as running, calisthenics, golf, gardening, or walking for exercise?”). The definition of *obesity* excluded the following: respondents measuring less than three feet or at least eight feet in height, weighing fewer than fifty pounds or 650 or more pounds, with a body mass index lower than 12 kg/m^2^ or 100 kg/m^2^ or higher, or who were pregnant. A total of fortyeight census tracts or neighborhoods were included in analyses with CDC health and behavioral outcomes.

Violence-related outcomes included the rate of domestic violence and violent crime calls to 911, with reports to follow. Domestic crime included calls for battery, assault, or disturbance believed to be between intimate partners, family members, or household members.^33^ The density measure at the neighborhood or tract level was built using 911 calls to the New Orleans Police Department for a domestic crime.^30^ For violent crime, a density measure was calculated based on 911 calls to the New Orleans Police Department for homicide or assault. Both measures were calculated per 1,000 population based on the population of the neighborhood or census tract.

The primary exposure of interest was the rate of police stop-and-frisk encounters or pat downs within a census tract per population in 2018, examined in total as well as separately for both encounters among Black residents and White residents and specifically for youth encounters (per youth population). The rate was categorized as high versus low based on the seventy-fifth percentile, given the lack of any consistent gold standard in categorization. The data were based on publicly accessible police stop-and-search field interviews, coded for mentions of pat-down for each search and by the race and age of the resident.

Key covariates at the neighborhood level include the Index of Concentration at the Extremes^34^ and concentrated disadvantage,^25^ used to capture the concentration of race and socioeconomic status in a given area, which may also be linked to frequent policing or perceived racial threat.^35,36^ We estimated the Index of Concentration at the Extremes for every census tract in New Orleans, using 2014–18 ACS five-year estimates of household income by race and ethnicity. Following the formula described by Nancy Krieger and colleagues, we calculated the Index of Concentration at the Extremes in each census tract by taking the difference between the number of non-Hispanic White families whose annual household income was greater than or equal to the eightieth percentile (≥$100,000) minus the number of non-Hispanic Black families whose household income was less than the twentieth percentile (<$25,000), divided by the total population in the tract with known income.^34^ Index values range from −1 (indicating that 100 percent of the population is concentrated in the most deprived group) to 1 (indicating that 100 percent of the population is concentrated in the most privileged group). Concentrated disadvantage was also measured using ACS 2018 five-year estimates to create an index based on six census variables: percentage of families with income below the poverty level, percentage of the population who self-identified as non-Hispanic Black, percentage of families receiving public assistance, percentage of female-headed households, percentage unemployed, and percentage younger than age eighteen. The percentages of each individual indicator were z-score transformed and then averaged into an overall index of concentrated disadvantage.

### Statistical Analysis

Descriptive bivariate and multivariate analyses were performed with SAS, version 9, as well as ArcPro, version 2.7.3, for spatial analyses. Multiple regression with generalized linear models in Proc Genmod was used to examine differences in health-related and violence-related outcomes by exposure to police encounters, accounting for sociodemographic characteristics of the neighborhoods, including concentrated disadvantage and racial and income residential segregation, as measured by the Index of Concentration at the Extremes. Spatial autocorrelation was also examined, and models were run with and without spatial weights. Spatial clustering was examined with Moran’s I to test spatial randomness.

### Limitations

Despite important findings, this study was not without its limitations. We relied on administrative, self-reported, and interpolated cross-sectional CDC survey data. However, self-reported measures of individual policing encounters were not available, and we were not able to examine health or violence outcomes by race, given the lack of disaggregated data. Our definition of *neighborhood* as a census tract was an additional limitation, given potential misclassification and theoretical operationalization of this administrative unit.^37^ Self-selection bias into neighborhoods is an additional possibility—and one that is difficult to account for, but more so without individual data available.We also did not examine other markers of frequent policing in the neighborhood, such as stop-and-frisk likelihood, nor did we have detailed data on the encounter itself, including the police involved or level of force. After we controlled for other neighborhood conditions, the substantive effects were greatly reduced. Some of this may be due to the difficulty in teasing out the relation between police encounters and characteristics of these neighborhoods, including potential mediating and overcontrol. Finally, given the cross-sectional nature of the data, the exact direction of these associations was difficult to tease out, and we were only able to observe spatial patterns and ecologic associations, some of which may be reciprocal in nature.

## Study Results

Characteristics of New Orleans census tracts are presented in exhibit 1. Nearly 20 percent of families lived below the poverty level, 15 percent of residents had less than a high school diploma, and the median annual household income was $46,492. However, neighborhoods were quite varied in socioeconomic and racial makeup, with concentrated disadvantage scores ranging from −1.98 to 2.77 (advantaged to disadvantaged) and Index of Concentration at the Extremes ranging from −0.80 to 0.70 (disadvantaged to privileged).

In terms of health and behavioral outcomes, neighborhoods can be characterized by high rates of smoking, poor health behaviors with regard to physical exercise and sleep, and very high rates of obesity and diagnosed diabetes. With respect to violence-related outcomes, we observed a violent crime rate of 20.87 per 1,000 (range: 0.00, 189.87) and a domestic violence call rate of 70.28 per 1,000 (range: 1.89, 636.36) (exhibit 1). Violent crime and domestic violence call rates were significantly correlated with the proportion of Black residents in the census tract (*r* > 0.50 for each; *p* < 0.0001) (data not shown).

As expected, the average rate of police stop-and-frisk encounters cited was more than three times higher for Black adults compared with their White counterparts (for example, total violation rate was 4.02 per 1,000 for Black adults versus 1.28 per 1,000 for White adults; *p* < 0.001). The overall rate of stop-and-frisk encounters in the neighborhoods was also high, at 5.47 per 1,000, but varied greatly across neighborhoods (range: 0.00, 101.93) (exhibit 1). Approximately 28 percent of neighborhoods were classified as having high (above the mean) rates both of stop-and-frisk encounters overall and of stop-and-frisk encounters for Black residents, given that the rate is primarily driven by encounters among Black residents (data not shown). Geographically, higher rates of stop-and-frisk encounters are concentrated in certain areas of the city, as shown in exhibit 2, with census tracts in red having higher rates and those in yellow having lower rates. There was substantial clustering by neighborhood in the overall rates of stop-and-frisk (Moran’s I = 0.061; *p* < 0.00001) and rate of stop-and-frisk among Black residents (Moran’s I = 0.068; *p* < 0.00001) (data not shown). These areas of the city are also largely those with more concentrated racialized income polarization based on the Index of Concentration at the Extremes, as shown in exhibit 3.

We observed a significant association between neighborhood stop-and-frisk encounters on both health and violence-related outcomes. As shown in exhibit 4 (crude models), compared to neighborhoods with lower rates of police encounters, those with high rates had significantly higher prevalence rates of smoking (5.43 percentage points), coronary heart disease (1.46 percentage points), obesity (6.04 percentage points), poor mental health (2.98 percentage points), poor physical health (3.57 percentage points), poor sleep (6.68 percentage points), and low physical activity (4.58 percentage points). However, neighborhoods with higher rates of police encounters had a significantly lower prevalence of self-reported diabetes diagnosis (−13.63 percentage points). Even after the Index of Concentration at the Extremes and concentrated disadvantage were controlled for (exhibit 4, adjusted models), associations remained significant for smoking, poor physical health, and physical activity in neighborhoods with higher versus lower numbers of police encounters. The observed crude inverse relation between encounter rate and diabetes diagnosis remained in the adjusted models, with a more than 5-percentage-point lower prevalence in higher-compared with lower-encounter neighborhoods. The lack of statistical significance for other health outcomes may also be a result of lower power given fewer census tracts in the CDC data (all *p* values <0.20).

There was a statistically significant association between the Index of Concentration at the Extremes and concentrated disadvantage on health outcomes, with the Index of Concentration at the Extremes being inversely and concentrated disadvantage positively associated with outcomes (data not shown).

All models run with the rate of stop-and-frisk among Black residents were virtually identical to those run with the rate overall, which was expected, given that the overall rate is driven primarily by rates among Black residents. Results were also similar both with and without spatial autocorrelation taken into account.

In terms of the association with violence, as shown in exhibit 4 (crude models), compared to neighborhoods with lower rates of police encounters, those with high rates experienced 21.53 more violent crime events and 70.49 more domestic violence events per 1,000; these differences were statistically significant (*p* < 0.05). After the Index of Concentration at the Extremes and concentrated disadvantage were controlled for, the association between high encounters and rates of violence was smaller in magnitude, although it remained high and statistically significant for violent crime and domestic violence, with 18.35 and 49.91 greater events per 1,000, respectively, in neighborhoods with high rates of police encounters.

## Discussion

We have added to the growing literature on policing in communities by including several health and behavioral as well as community violence markers and by quantifying the association in New Orleans at the neighborhood level and in dense, urban areas with a history of violence and overusage of the criminal legal system. The results suggest a spatial patterning of police encounters in this southern city and a significant relation between frequent policing and rates of smoking, inadequate physical activity, and poor self-reported physical health, as well as a marginal association with obesity, coronary heart disease, poor mental health, and poor sleep. We also observed a significant, positive association between frequent policing and neighborhood rates of violent crime and domestic violence and an inverse relation between frequent policing and self-reported diabetes diagnosis.

Our results corroborate those of previous studies to some extent, with a positive association between stop-and-frisk and self-reported poor mental health and poor sleep, as in several published studies on police interactions and mental health^15^ and sleep quantity and quality.^38,39^ Neither association remained significant in adjusted models, perhaps given low power resulting from a small number of census tracts. And similar to an analysis of New York City stop-and-frisk data by Abigail Sewell and Kevin Jefferson,^17^ we also observed mixed evidence with regard to the association between frequent policing and health outcomes.

Although the link between frequent policing and violence-related outcomes has been theoretically studied, empirical tests of these relations are limited. Whereas the relationship between neighborhood violent crime and domestic violence and policing is complex, the potential breakdown of social cohesion and weaker ties could lead to increased rates of violent crime and domestic events^25,40,41^ and may also be contributing to the racial disparities seen in both violent crime and domestic violence.^42,43^

This study adds to the growing evidence base estimating relationships between frequent policing—a potential indicator of structural racism^2,44,45^—and health and well-being, including domestic violence. Given the limited number of studies examining physical health outcomes of frequent policing, despite the potential impact of such stress on physiologic markers^23,46^ and risk for cardiovascular and other diseases,^22^ this is one contribution to the much-needed empirical evidence in this space. Although examination of the association by race was not possible with available data, our findings contribute to our stated hypothesis that frequent policing in Black communities may be associated with racial health disparities across many communities.^2,17,24^

Whereas greater community violence may increase police surveillance in areas and contribute to the cycle of frequent policing and subsequent violence, there are alternatives that should be considered. A recent report by the John Jay College of Criminal Justice^47^ outlines several strong theoretically and empirically based alternatives to frequent policing that may be efficacious at reducing violence in US communities, including changes in physical characteristics of communities such as removing vacant and blighted property, strengthening antiviolence norms and peer relationships, increased youth support, mitigating financial stress, addressing the harmful effects of the justice process, and policies that control access to and possession of firearms. Some of these strategies may be easier than others, although all have implications not only for reduced community violence but also for health and well-being. There is a great need for strengthened policy and programming focused on the role of frequent policing on health and violence outcomes and its role in racial health inequities.^48^

## Figures and Tables

**EXHIBIT 2:**
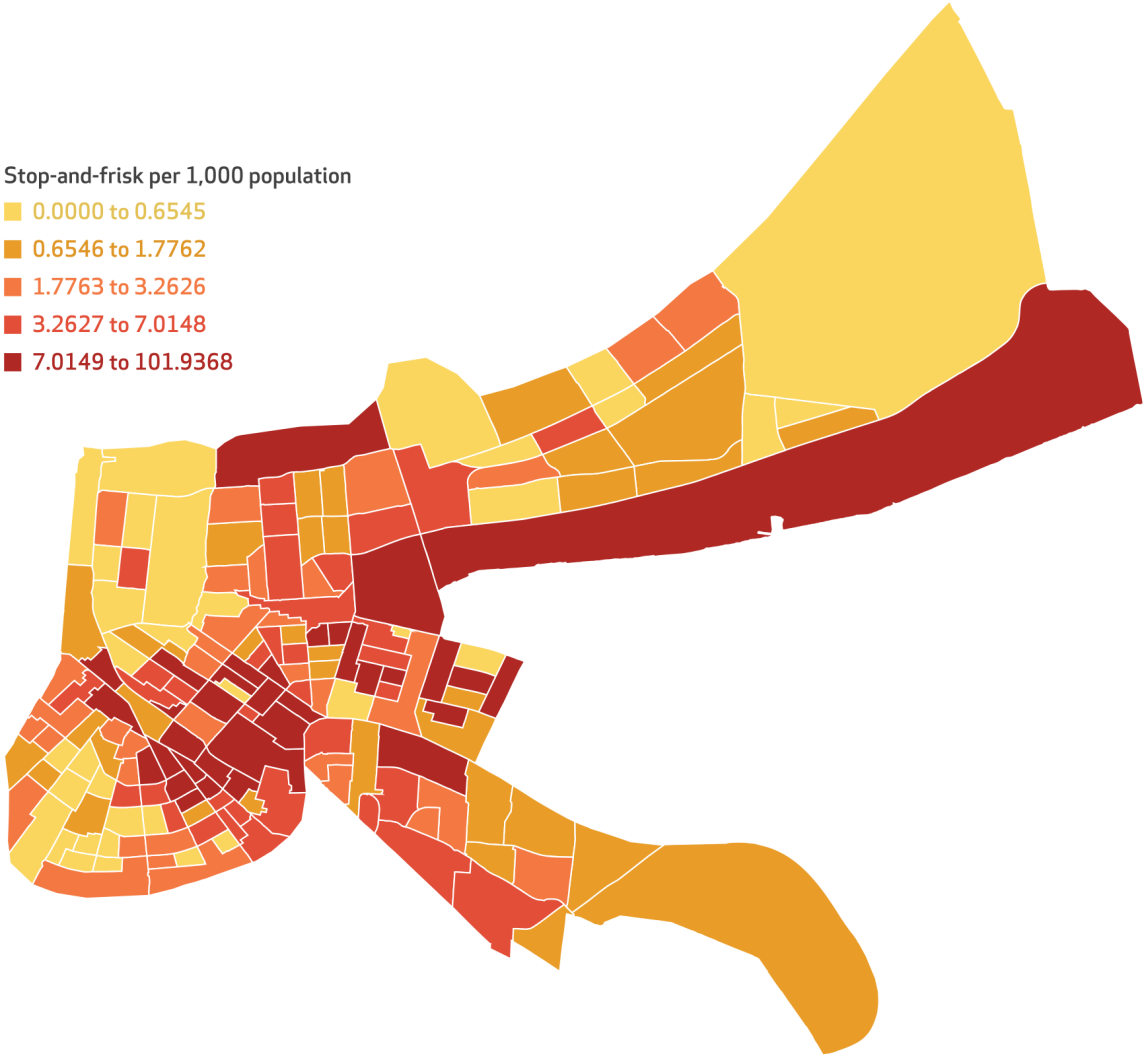
Rates of police stop-and-frisk encounters across neighborhoods in New Orleans, Louisiana, per 1,000 population, 2018 **SOURCE** City of New Orleans. **NOTE** Neighborhoods correspond to census tracts.

**EXHIBIT 3:**
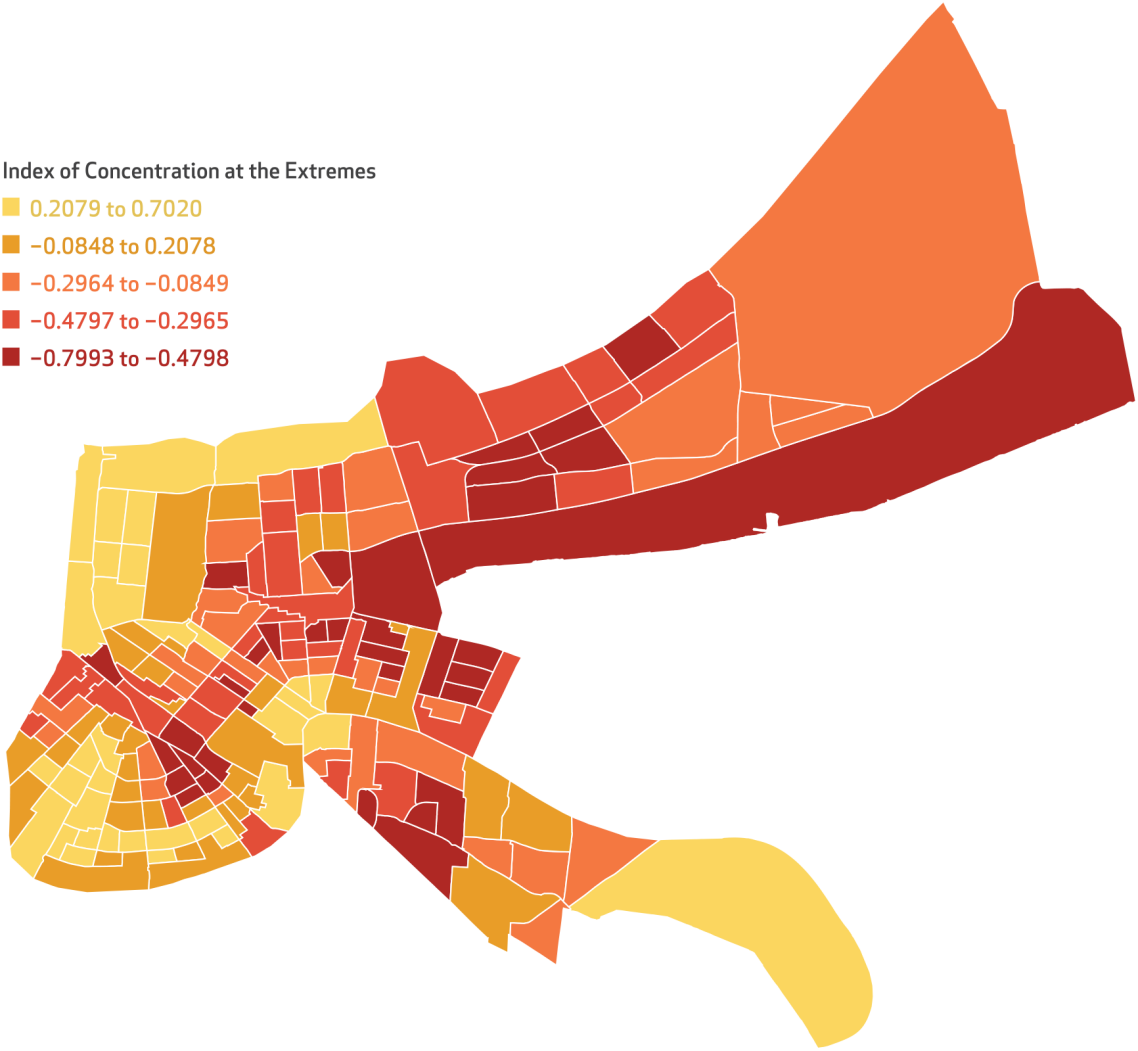
Index of Concentration at the Extremes in New Orleans, Louisiana, by neighborhood, 2014-18 **SOURCE** Census Bureau, American Community Survey data. **NOTES** Values of the Index of Concentration at the Extremes range from −1 (indicating that 100 percent of the population is concentrated in the most deprived group) to 1 (indicating that 100 percent of the population is concentrated in the most privileged group). Neighborhoods correspond to census tracts.

**EXHIBIT 1:** Characteristics of census tracts in New Orleans, Louisiana, 2018

Variables	Mean	SD	Range
**DEMOGRAPHIC CHARACTERISTICS** ^a^			
Median household income ($)	46,492.47	30,877.09	10,519–172,727
Families on public assistance (%)	20.20	15.39	0.00–70.68
Families living below poverty level (%)	19.66	16.04	0.00–77.35
Unemployed (%)	9.03	5.81	0.00–35.50
Less than high school diploma (%)	15.10	4.07	0.00–54.90
Female head of household (%)	48.47	11.02	0.00–100.00
Self-identified as non-Hispanic Black (%)	57.11	33.15	0.00–99.13
Index of Concentration at the Extremes, disadvantaged to privileged	−0.15	0.35	−0.80–0.70
Concentrated disadvantage (z-score), advantaged to disadvantaged	−0.02	0.84	−1.98–2.77
**HEALTH AND BEHAVIORAL CHARACTERISTICS** ^b^			
Current cigarette smoker (%)	21.69	7.90	11.20–45.90
Coronary heart disease (%)	6.38	2.17	3.30–11.10
Obesity (%)	34.55	9.06	23.00–56.00
Poor mental health (%)	15.55	4.81	9.30–32.30
Poor physical health (%)	14.64	5.63	7.90–32.40
Poor sleep (<7 hours per night) (%)	39.52	6.90	29.20–53.70
Lack of leisure time physical activity (%)	28.95	10.13	15.90–54.40
Diagnosed diabetes (%)	57.97	16.28	22.10–80.10
**VIOLENCE** ^a^			
Violent crime rate per 1,000	20.87	13.75	0.00–189.87
Domestic violence call rate per 1,000	70.28	85.18	1.89–636.36
Community policing			
Sbottom-and-frisk rate per 1,000			
Total	5.47	11.55	0.00–101.93
Black	4.02	7.17	0.00–101.93
White	1.28	4.51	0.00–42.30
Youth sbottom-and-frisk rate per 1,000			
Total	1.27	5.67	0.00–36.00
Black	1.27	5.67	0.00–36.00
White	0.00	0.00	0.00–0.00

**SOURCES** Census Bureau, American Community Survey; Centers for Disease Control and Prevention (CDC) 500 Cities Project; and City of New Orleans.

a *N* = 174 census tracts.

b *N* = 48 census tracts, given available CDC 500 Cities Project data for census tracts in New Orleans.

**EXHIBIT 4:** Association between police encounters (high versus low) and health and violence outcomes: results from crude and adjusted regression models, New Orleans, Louisiana, 2016-19

	Crude estimates		Adjusted estimates^a^	
Outcomes	Beta estimate	*p* value	Beta estimate	*p* value
**HEALTH AND BEHAVIORAL CHARACTERISTICS** ^b^				
Current cigarette smoker (%)	5.43	0.02	1.90	0.01
Coronary heart disease (%)	1.46	0.02	0.55	0.14
Obesity (%)	6.04	0.03	1.50	0.06
Poor mental health (%)	2.98	0.04	0.99	0.05
Poor physical health (%)	3.57	0.04	1.19	0.04
Poor sleep (<7 hours per night) (%)	6.68	0.03	0.90	0.16
Lack of leisure time physical activity (%)	4.58	0.03	1.76	0.04
Diagnosed diabetes (%)	−13.63	0.001	−5.42	0.0004
**VIOLENCE** ^c^				
Violent crime rate per 1,000	21.53	<0.0001	18.35	<0.0001
Domestic violence call rate per 1,000	70.49	<0.0001	49.91	<0.0001

**SOURCES** Census Bureau, American Community Survey; Centers for Disease Control and Prevention (CDC) 500 Cities Project; and City of New Orleans. **NOTE** Although the health and behavioral variables are percentages, the beta estimates show percentage-point differences.

a Adjusted for Index of Concentration at the Extremes and concentrated disadvantage.

b CDC 500 Cities Project data only included 48 census tracts in New Orleans.

c *N* = 174 census tracts.
